# Supplementary material for: Glioblastoma stem cells show transcriptionally correlated spatial organization
Source: Commun Biol. 2026 Jan 23;9:208. doi: 10.1038/s42003-026-09566-2 (PMC12894897; doi:10.1038/s42003-026-09566-2)
Supplement: Supplementary file 2 — Supplementary Information [file 42003_2026_9566_MOESM2_ESM.pdf]

SUPPLEMENTARY FIGURES 1-6

Glioblastoma stem cells show transcriptionally correlated spatial organization

Shamini Ayyadhury, Patty Sachamitr, Michelle M. Kushida, Nicole I Park, Fiona J. Coutinho, Owen Whitley, Panagiotis Prinos, Cheryl H. Arrowsmith, Peter B. Dirks, Trevor J. Pugh, Gary D. Bader

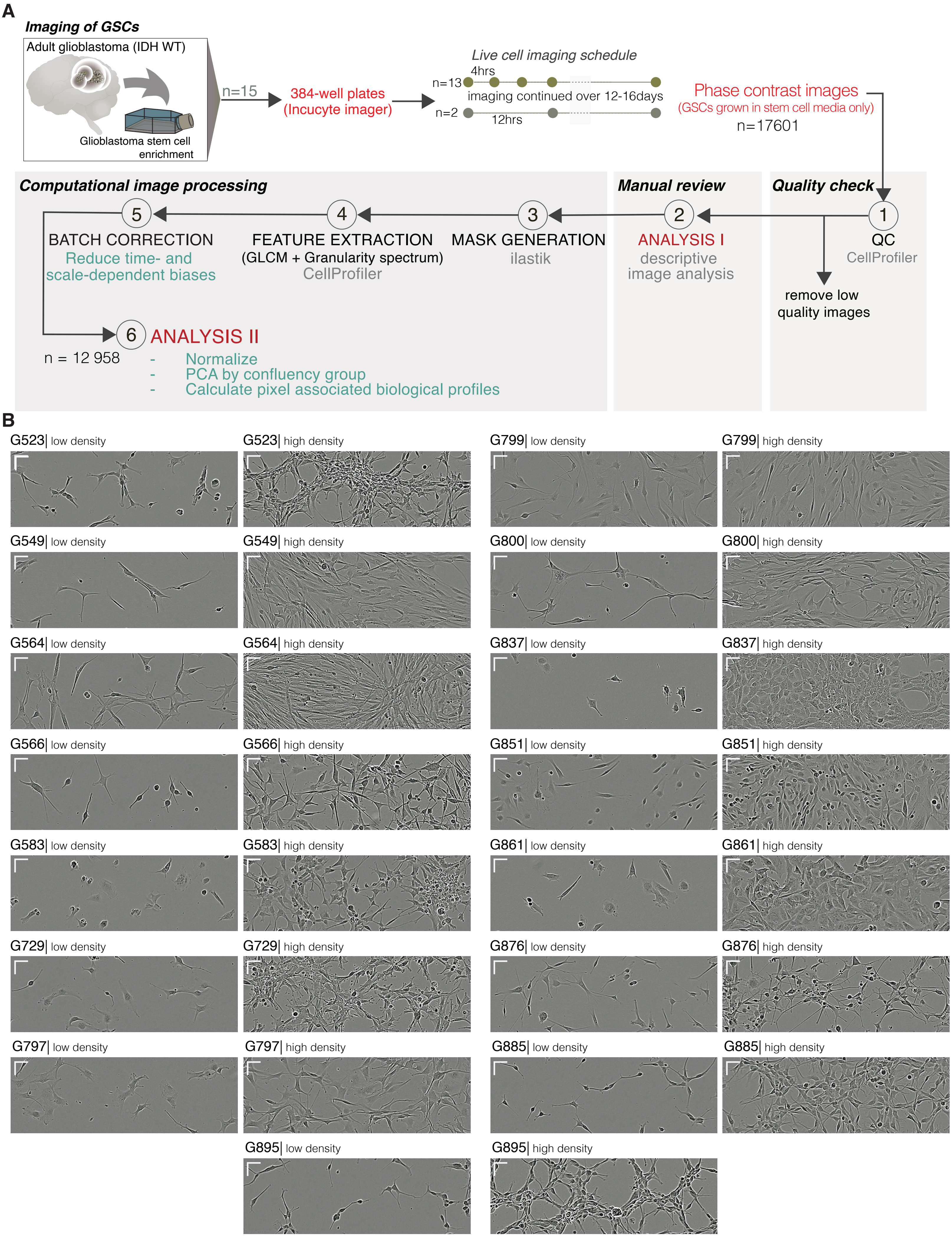

Supplementary figure 1. Analysis workflow and representative images from our 15 GSC samples. A) Workflow for manual and computational analysis and B) representative images from 15 patient-derived GSCs from low and high-density phase-contrast images. Scale bar = 50um for both x and y-axis bars.

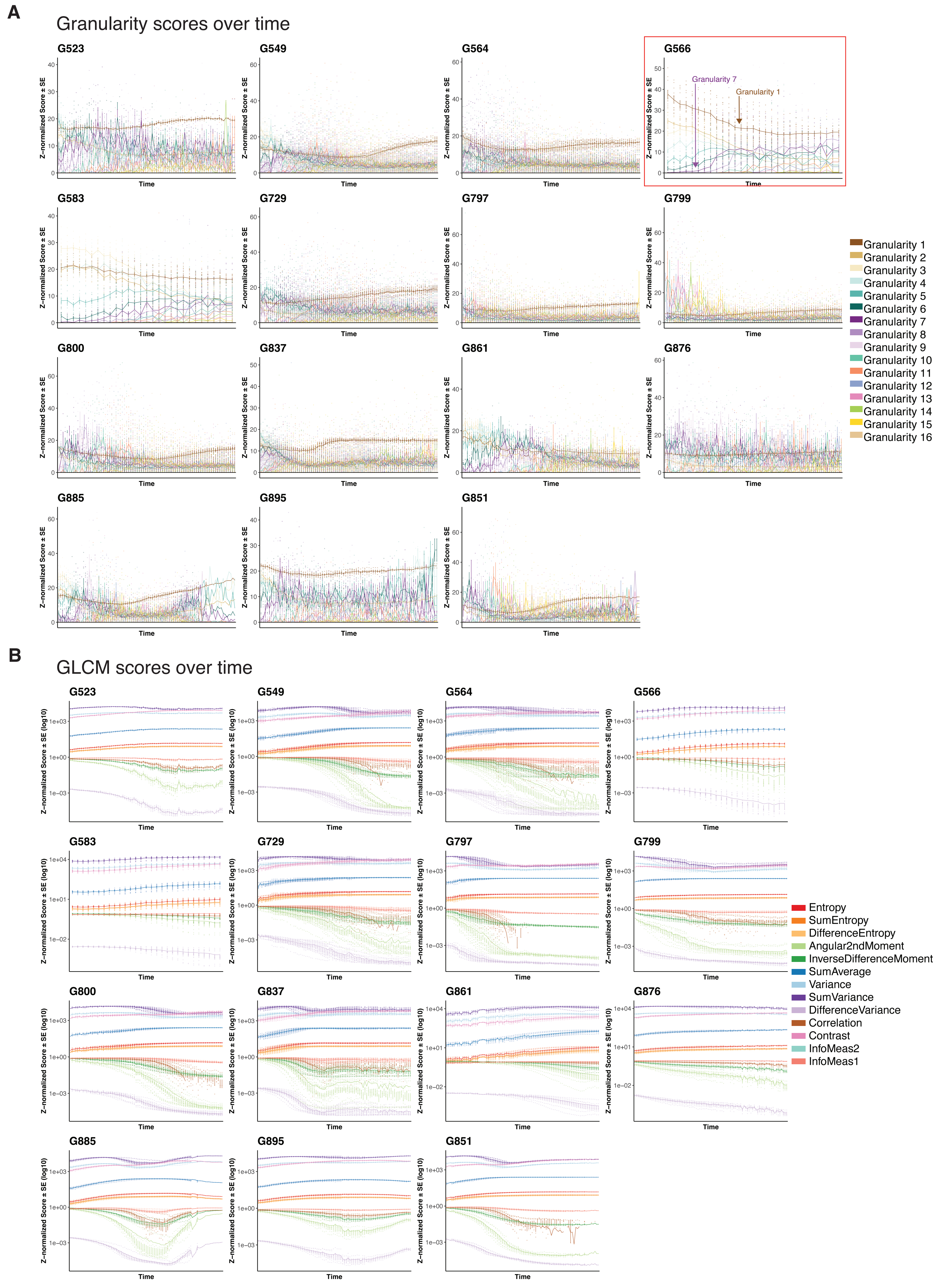

Supplementary figure 2. Multicellular patterning of GSCs in culture across time and space. By using CellProfiler to profile each image using 29 statistical features for pixel intensity distributions and pixel-pixel spatial variational patterning, we can quantify the variation in pixel spatial organization across time and space. A) Granularity and B) GLCM textural features (y-axis) are plotted across time-points (x-axis) as cells are growing on a 2D surface. Red box in A) highlights an example to show how two features (Granularity 1 and 7 show opposing patterns across time, reflecting the changes in collective organization highlighted in Supplementary fig. 1. y-axis scale: Granularity scores are z-normalized values whereas GLCM scores are represented on a log10 scale after z-normalization. x-axis represents imaging time points over cell growth. Error bars represent mean ± standard error of the mean (SE) for each feature per time-point. Sample size : n=15.

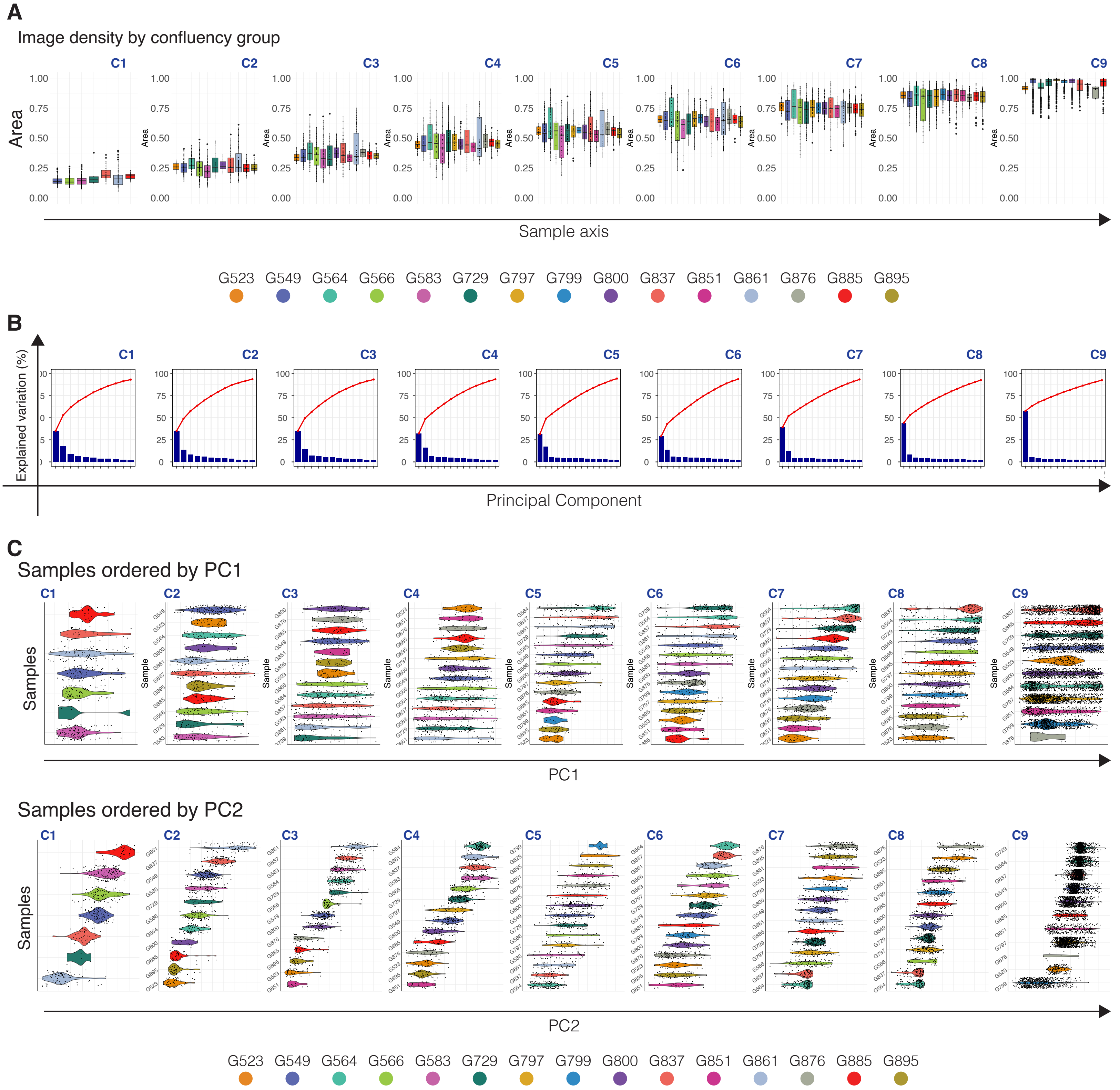

Supplementary figure 3. Pattern recognition across confluency levels. A) Cell density variation for all images across samples for each of nine confluency levels. y-axis represents cell density and x-axis represents samples. One panel is shown for each confluency level, with confluency level 1 (lowest confluency) on the left panel and confluency level 9 (highest confluency) on the right. Boxplots show the median and interquartile range; individual points represent single observations. B) Principal component explained variance for nine confluency levels. y-axis shows percent explained variance per principal component (x-axis) for 12 components. One panel is shown for each confluency level, with confluency level 1 (lowest confluency) on the left panel and confluency level 9 (highest confluency) on the right. C) Mean sample PC1 and PC2 scores shown for all confluency levels, with samples in each level ordered by the sample mean of the corresponding PC. y-axis shows samples and x-axis shows mean PC value. One panel is shown for each confluency level, with confluency level 1 (lowest confluency) on the left panel and confluency level 9 (highest confluency) on the right. D) Schematic of correlation analysis performed between PCA of image features and gene signatures from bulk RNA expression after PC directional correction (see materials and methods). The correlation between the mean sample PC scores and each of the 111 gene signatures (s1-s111) was computed for all nine confluency levels (C1-C9). Confluency group size: C1:n=7, C2: n=11, C3: n=13, C4: n=14, C5: n=15, C6: n=15, C7: n=14, C8: n=13, C9: n=11.

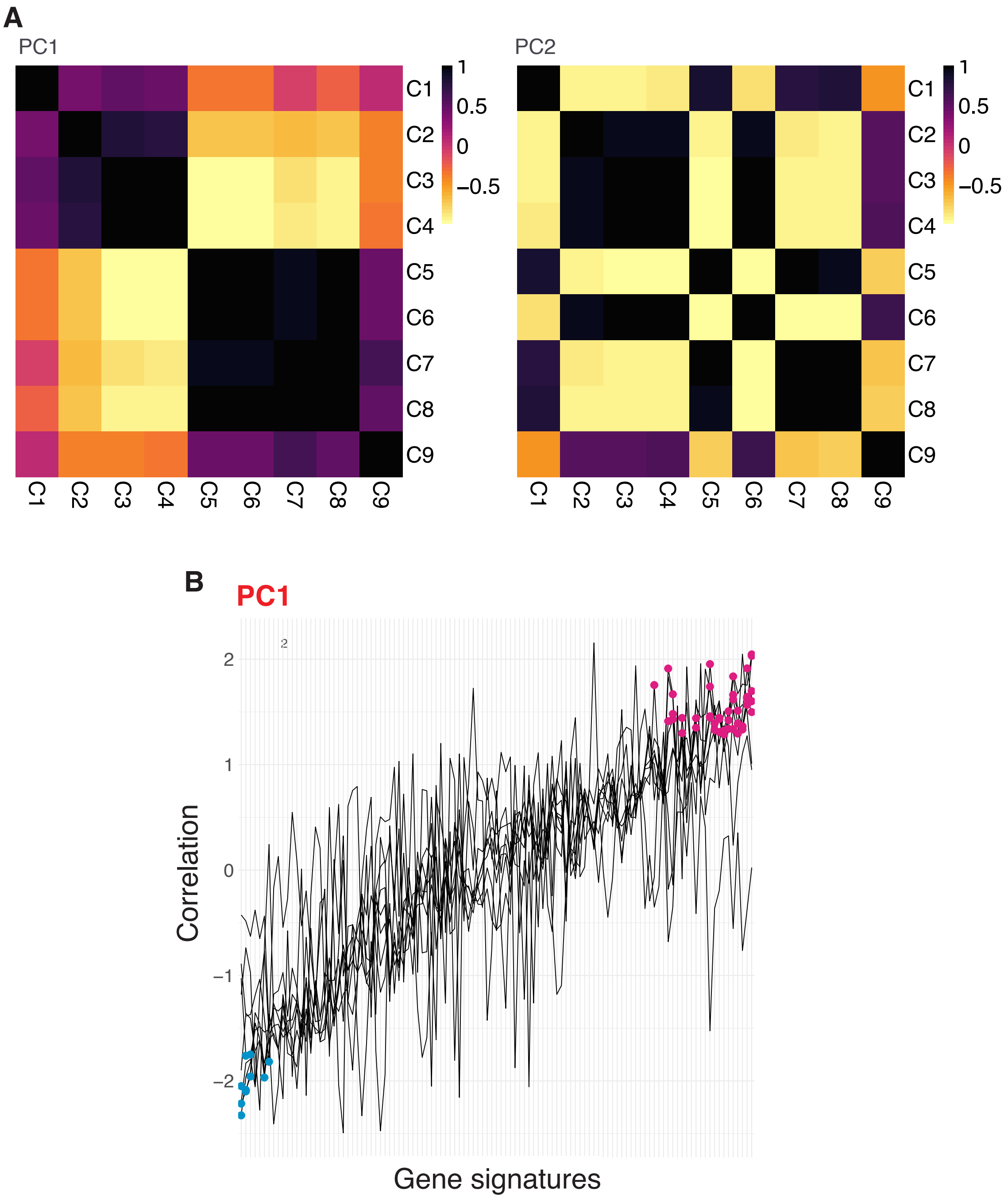

Supplementary figure 4. Evaluating PC1 and PC2 relationship with gene signature scores. A) Matrices of correlation between confluency levels for PC1 and PC2 (after directional correction to align PC1 and PC2). Dark color represents high correlation and light color represents anti-correlation. Confluency groups : n=9. B) The same plot as Figure 2B, but for PC1, showing the same overall trend. PC1 correlations between image-derived mean-PC1 scores and the GSVA scores for matched samples for each of the 111 gene signatures for all nine confluency levels. Each line represents a confluency level. The y-axis represents the pearson correlation between the mean-PC1 vectors for 15 samples and the GSVA scores derived from matched samples, for each of the 111 gene signatures from bulk gene expression datasets. The x-axis represents 111 gene signatures used to compute the GSVA scores and ordered by the correlation values. Gene signatures with statistically significant correlations ($p < 0.05$) are colored by the major biological category they represent (magenta=neurodevelopmental and blue=mesenchymal, injury-response). Gene signatures shown : n=111.

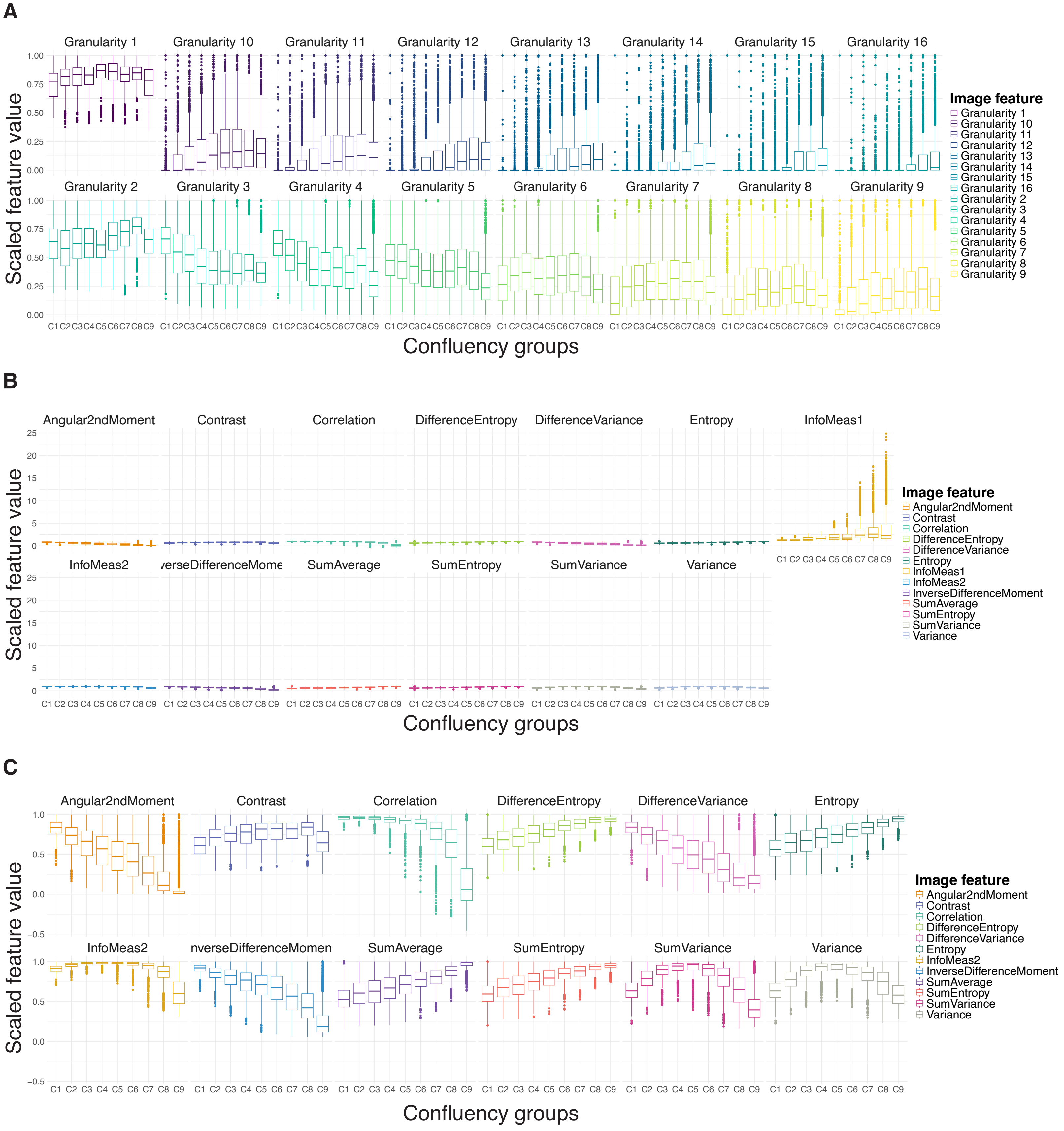

Supplementary figure 5. Image features show broad patterns of variation along cell density levels. A) The variation in granularity spectrum across different cell densities. B) GLCM features vary along confluency levels, with features belonging to the same or similar feature families following the same pattern. C) Same as B) but after removing Informational Measure 1 as an outlier. For all panels, y-axis is the scaled image feature value obtained by dividing the feature vector by the maximum feature value and x-axis represents the confluency levels from C1 (lowest confluency) to C9 (highest confluency). Confluency groups: n=9 for each image feature. Boxplots show the median and interquartile range; individual points represent single observations.

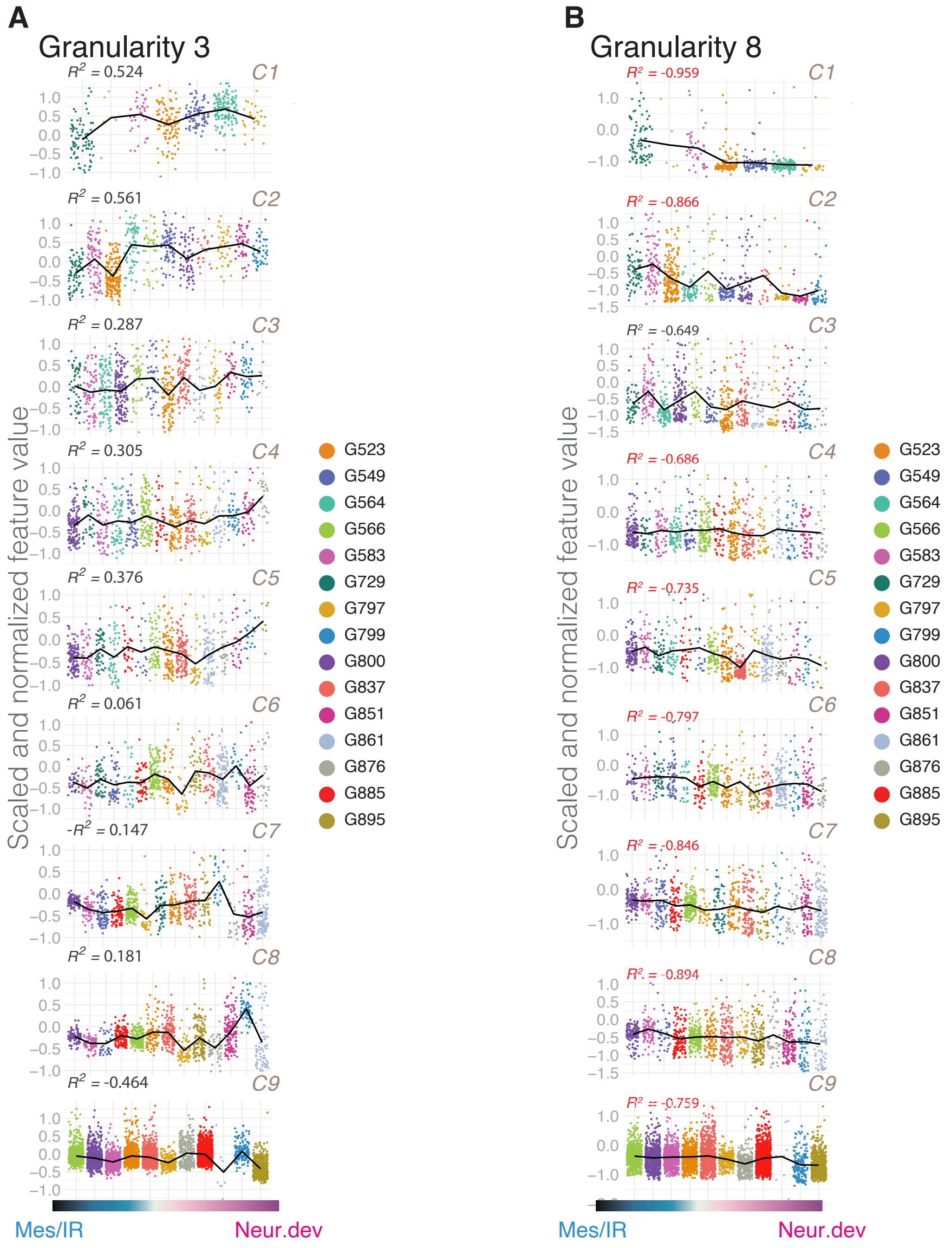

Supplementary figure 6. Representative image features correlated with the GSC transcriptional gradient. A) Granularity 3 follows the GSC gradient better at lower confluency levels, with higher feature values associated with neurodevelopmental GSC images, indicating smaller structural units or patches characteristic of these samples, whereas B) mesenchymal/injury response images show larger patches or clusters of cells at lower densities as indicated by the higher signals from the mid granularity spectrum (shown here using a representative example feature of Granularity 8). For all panels, the y-axis represents the scaled and normalized values for each feature represented in the panel (see materials and methods). Samples are ordered along the x-axis by their mean PC2 value. Images from each sample are colored separately.For all panels, the R2 value is colored red if p < 0.01. Biologically independent sample size within each confluency group: C1:n=7, C2: n=11, C3: n=13, C4: n=14, C5: n=15, C6: n=15, C7: n=14, C8: n=13, C9: n=11.
