# Supplementary material for: Glioblastoma stem cells show transcriptionally correlated spatial organization
Source: Commun Biol. 2026 Jan 23;9:208. doi: 10.1038/s42003-026-09566-2 (PMC12894897; doi:10.1038/s42003-026-09566-2)
Supplement: Supplementary file 3 — Supplementary Information [file 42003_2026_9566_MOESM3_ESM.docx]

**SUPPLEMENTARY NOTES 1-2**

**Glioblastoma stem cells show transcriptionally correlated spatial organization**

Shamini Ayyadhury, Patty Sachamitr, Michelle M. Kushida, Nicole I Park, Fiona J. Coutinho, Owen Whitley, Panagiotis Prinos, Cheryl H. Arrowsmith, Peter B. Dirks, Trevor J. Pugh, Gary D. Bader

**Supplementary note 1**

1. **Understanding GLCM computation**
   1. **Toy Example Image**

Each image is defined by a matrix of pixels. The range of each pixel is defined by the bit size. (This is in contrast to the gray level co-occurrence matrix which is symmetric and the rows and columns are defined by the pixel bit value (i.e 8 bit = 256 rows/columns))

**

**

**Supplementary note fig 1.** 6 pixel x 6 pixel.

- 1. **Construction of the GLCM**

The figure below will explain step by step the construction of the GLCM and the subsequent mathematical derivatives.

**Figure 1A.** The GLCM model can be constructed from using the entire image or a window can be designated to analyze a portion of an image. Here we generate a gray level co-occurrence matrix for a window (15 pixel x 15 pixel) using a sample image (G564 grown to high confluency).

**Figure 1B.** This is the 15 x 15 image pixel matrix.

**Figure 1C**. First a 256 x 256 matrix is constructed. The dimensions are determined by the pixel bit and in this example we are analyzing a 8 bit image which generates 256 gray levels.

Starting from the left and moving towards the right, the pixel on the left serves as a reference pixel and the pixel on the right the neighbor pixel. Once reaching the rightmost column, the process is repeated from right to left, where the right pixel serves as the reference pixel and the left pixel serves as the neighbor pixel.

In this case, the original 15 x 15 image window has only 1 (133,135) pixel pair in the forward (left to right) direction but 2(133,135) pixel pairs in the backward (right to left) direction. We tabulate the counts for these pixel pairs.

**Figure 1D**. We obtain the final horizontal matrix by adding the forward (A) and backward (B) matrices. Note that B = A^T^, therefore we can simplify this step by computing only the counts matrix for one direction and adding the transpose to it.

GLCM is constructed in 4 main directions - horizontal, vertical and 2 diagonal. We show the horizontal construction as an example.

**Figure 1E.** The matrix is divided by the total number of counts calculated to derive a probability distribution matrix which we call the normalized GLCM.

**Figure 1F.** This probability matrix is used to evaluate the spatial pixel distribution patterns found in an image.


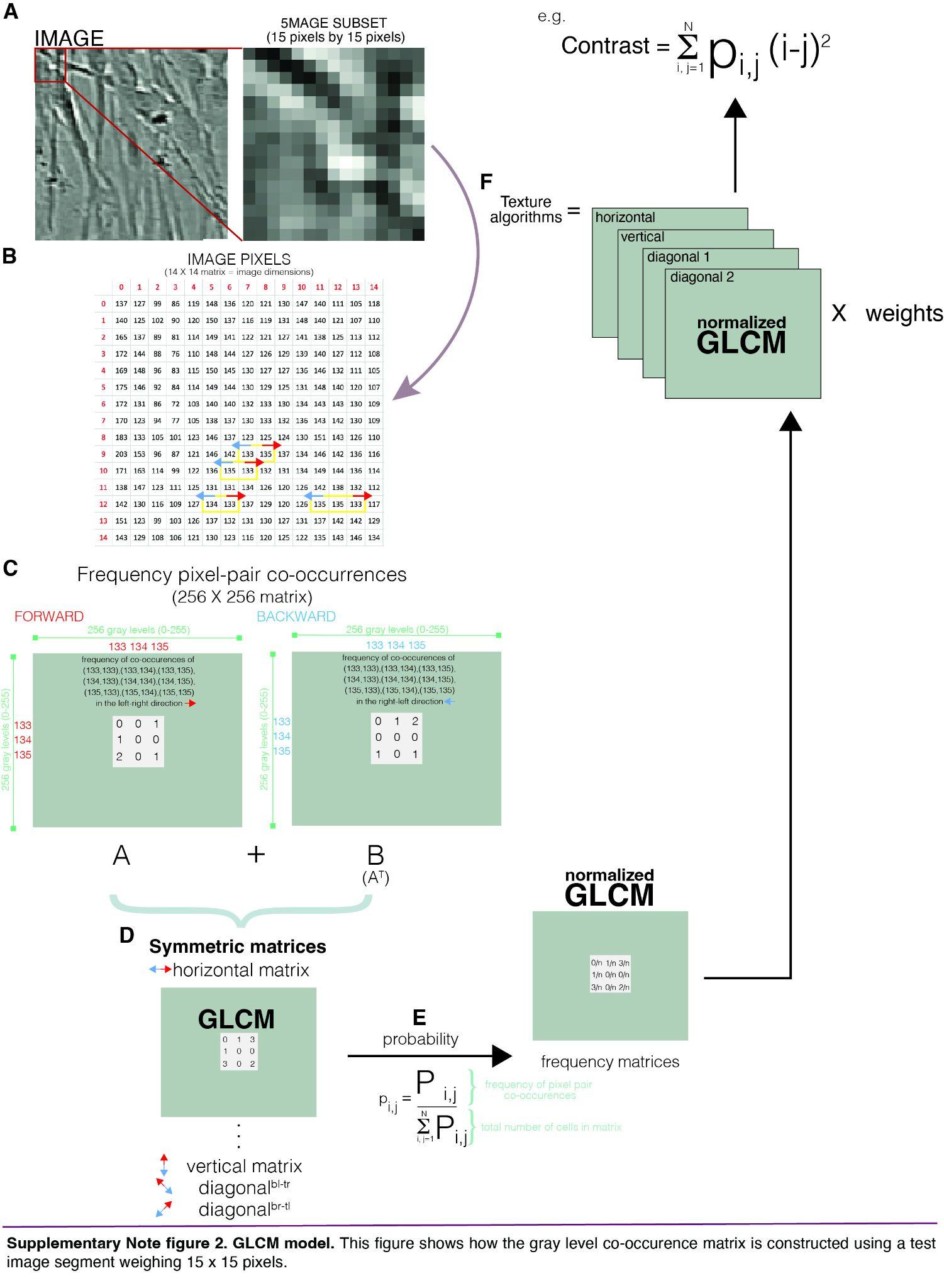


- 1. **GLCM can be constructed using different scale factors**

Reference-Neighbour pixel pairs can take on different range or scales as shown below

**

**

**Supplementary note fig 3.** Scales in GLCM construction. For scale =1, the reference pixel and neighbor pixel are adjacent, whereas for scale=2, the neighbor pixel is 2 pixels away.

For our example below, we will use scale=1.

- 1. **GLCM-derived mathematical algorithms**

**Legend**

$Ng = gray levels (pixel bit)$

The scale factor determines the distance between the reference and neighbor pixels that’s used to compute the GLCM.

$p_{x}(i) = ith entry in the marginal probability matrix = \sum_{i=1}^{Ng} p(i,j)$

$p_{y}(j) = jth entry in the marginal probability matrix = \sum_{j=1}^{Ng} p(i,j)$

$p_{x+y}(i+j) = probability distribution of the sum of two gray levels = \sum_{i=0}^{Ng} \sum_{j=0}^{Ng} p(i,j)$

$i+j \in[0,1,...,1Ng] x \& y represent gray levels not coordinates$

$p_{x-y}(i+j) = probability distribution of the difference of two gray levels = \sum_{i=0}^{Ng} \sum_{j=0}^{Ng} p(i,j)$

|$i-j\vert\in[0,1,...,Ng] x \& y represent gray levels not coordinates$

$\mu_{x} , \mu_{y} , \sigma_{x} ,\sigma_{y} are the main standard deviations of p_{x} and p_{y}$

Gray level co-occurrence matrix is constructed from the pixels of images from the normalized GLCM.

GLCM (normalized)

$p(i,j) = \frac{P(i,j)}{\sum_{i=1}^{Ng} \sum_{j=1}^{Ng} P(i,j)}$

**GLCM derived hand-engineered pixel features**

1. Contrast

Computes a larger weight when the difference between the reference and

neighbour pixel is higher and hence results in a larger output when the image is

dominated by sharp variations in pixel values.

$\sum_{i=1}^{Ng} \sum_{j=1}^{Ng} {(i-j)}^{2}{p(i,j)}$

1. Inverse difference moment

Measures the homogeneity of an image window. The higher the value, the more homogeneous the gray level pixel-pairs are in the area inspected. Here pixel pairs moving away from the diagonal (and hence having more contrast) reduce the homogeneity value and vice versa.

$\sum_{i=1}^{Ng} \sum_{j=1}^{Ng} \frac{p(i,j)}{1 + (i-j)2}$

1. Angular Second Moment (ASM)

The ASM is a measure of the homogeneity as well, except that it is simply the

square of the normalized GLCM and effectively gives equal weight to all pixel pair differences as opposed to IDM where pixel pairs with larger contrast are penalized more.

$\sum_{i=1}^{Ng} \sum_{j=1}^{Ng} {p{(i,j)}^{2}}$

1. Sum of squares (Variance)

Computes the variance in the reference pixels and their weighted contribution towards the GLCM frequency distribution and sums it across all gray level pixel pairs. A higher value means the presence of higher contribution of pixel pairs with larger pixel value differences. Related to Contrast.

$\sum_{i=1}^{Ng} \sum_{j=1}^{Ng} {(i-\mu)}^{2}{p(i,j)}$

1. Sum Variance

Variance of sum of gray levels

$\sum_{i=2}^{2(Ng)} {(i-Sum Average)}^{2}{p_{x+y}(i)}$

1. Difference Variance

Variance of difference of gray levels

$\sum_{i=1}^{Ng} {(i-\mu)}^{2}{p_{x-y}(i)}$

1. Entropy

Measure of randomness of intensity values in an image

$-\sum_{i=1}^{Ng} \sum_{j=1}^{Ng} p(i,j) log(p(i,j))$

1. Sum Entropy

Measure of randomness of sum of gray level intensity values in an image

$-\sum_{i=1}^{2(Ng)} p_{x+y}(i) log(p_{x+y}(i))$

1. Difference Entropy

Measure of randomness of difference in gray level intensity values in an image

$-\sum_{i=1}^{Ng} p_{x-y}(i) log(p_{x-y}(i))$

1. Sum Average

The average sum of gray levels

$\sum_{i=2}^{2Ng} {ip}_{x+y}(i)$

1. Correlation

The correlation between gray-level pixel pairs from a GLCM.

$\sum_{i=1}^{Ng} \sum_{j=1}^{Ng} \frac{(i\cdot j)p(i,j) - \mu_{x}\mu_{y}}{\sigma_{x}\sigma_{y}}$

1. Information measures of correlation (IMC)

IMC1

$\frac{Entropy - HXY1}{max(HX, HY)}$

$HX = -\sum_{i}^{Ng} p_{x}(i)log(p_{x}(i)) = entropy of p_{x}$

$HY = -\sum_{j}^{Ng} p_{y}(j)log(p_{y}(j)) = entropy of p_{y}$

$HXY1 = -\sum_{i}^{Ng} \sum_{j}^{Ng} p(i,j)log[p_{x}(i)p_{y}(j)]$

IMC2

$[1- exp(-2(HXY2 - Entropy)]^{1/2}$

$HXY2 = -\sum_{i}^{Ng} \sum_{j}^{Ng} p_{x}(i)p_{y}(j)log[p_{x}(i)p_{y}(j)]$

**Supplementary note 2**

**Granularity spectrum**

Granulometry computes the size distribution by using a series of morphological operators (functions). The granularity spectrum used in this study uses a range of structuring elements varying in width from 1 pixel to 16 pixels. Each structuring element applied over the image will remove pixels that are equal or smaller than itself. The differences between the proportion of pixels removed in successive structuring element applications produces the size distribution inherent in the image.

**References**

[1. Esteban, Á. E. et al. A new optical density granulometry-based descriptor for the classification of prostate histological images using shallow and deep Gaussian processes. Comput. Methods Programs Biomed. 178, 303–317 (2019).](https://sciwheel.com/work/bibliography/14764235)
