# Supplementary material for: Glioblastoma stem cells show transcriptionally correlated spatial organization
Source: Commun Biol. 2026 Jan 23;9:208. doi: 10.1038/s42003-026-09566-2 (PMC12894897; doi:10.1038/s42003-026-09566-2)
Supplement: Supplementary file 4 — Reporting Summary [file 42003_2026_9566_MOESM4_ESM.pdf]

nature portfolio

Corresponding author(s): SHAMINI AYYADHURY

Last updated by author(s): Dec 24, 2025

Reporting Summary

Nature Portfolio wishes to improve the reproducibility of the work that we publish. This form provides structure for consistency and transparency in reporting. For further information on Nature Portfolio policies, see our Editorial Policies and the Editorial Policy Checklist.

Statistics

For all statistical analyses, confirm that the following items are present in the figure legend, table legend, main text, or Methods section.

n/a	Confirmed	
☐	☒	The exact sample size (*n*) for each experimental group/condition, given as a discrete number and unit of measurement
☐	☒	A statement on whether measurements were taken from distinct samples or whether the same sample was measured repeatedly
☐	☒	The statistical test(s) used AND whether they are one- or two-sided<br>*Only common tests should be described solely by name; describe more complex techniques in the Methods section.*
☒	☐	A description of all covariates tested
☒	☐	A description of any assumptions or corrections, such as tests of normality and adjustment for multiple comparisons
☐	☒	A full description of the statistical parameters including central tendency (e.g. means) or other basic estimates (e.g. regression coefficient) AND variation (e.g. standard deviation) or associated estimates of uncertainty (e.g. confidence intervals)
☒	☐	For null hypothesis testing, the test statistic (e.g. *F*, *t*, *r*) with confidence intervals, effect sizes, degrees of freedom and *P* value noted<br>*Give P values as exact values whenever suitable.*
☒	☐	For Bayesian analysis, information on the choice of priors and Markov chain Monte Carlo settings
☒	☐	For hierarchical and complex designs, identification of the appropriate level for tests and full reporting of outcomes
☐	☒	Estimates of effect sizes (e.g. Cohen's *d*, Pearson's *r*), indicating how they were calculated

Our web collection on statistics for biologists contains articles on many of the points above.

Software and code

Policy information about availability of computer code

Data collection	All phase contrast images were captured and collated using Incucyte ZoomTM live cell imaging system (Essen Biosciences)
Data analysis	ilastik(v1.4.0), CellProfiler(v4.0.7) were used to generate image masks and extract image features from all images.<br>All data processing and statistical analyses were conducted in R (4.4.2) within an active renv environment to support reproducibility. Major Bioconductor and CRAN packages used BiocManager (1.30.25), SummarizedExperiment (1.36.0); GSVA (2.0.6) and GSEABase (1.68.0) for gene-set variation analysis; PCAtools (2.18.0) for principal-component computations and visualization; glmnet (4.1-8) for ridge and elastic-net regression; dplyr (1.1.4) and tidyr (1.3.1) for data wrangling; reshape2 (1.4.4) for reshaping data matrices; ggplot2 (3.5.1), ggpubr (0.6.0), and ggbiplot (0.6.2) for data visualization and figure assembly; and pheatmap (1.0.12) for heatmap generation. Each package version was recorded in the project's renv.lock file to support consistency across analyses.

For manuscripts utilizing custom algorithms or software that are central to the research but not yet described in published literature, software must be made available to editors and reviewers. We strongly encourage code deposition in a community repository (e.g. GitHub). See the Nature Portfolio guidelines for submitting code & software for further information.

Data

Policy information about availability of data

All manuscripts must include a data availability statement. This statement should provide the following information, where applicable:

- Accession codes, unique identifiers, or web links for publicly available datasets
- A description of any restrictions on data availability
- For clinical datasets or third party data, please ensure that the statement adheres to our policy

Images and related data can be found on Zenodo via a unique DOI : 10.5281/zenodo.18046405
Previously published bulk RNA-seq data that were re-analyzed in this study are available from the following sources - EGAS00001003070 and EGAS00001004395 through the European Genome-Phenome Archive repository in the form of FASTQ or BAM files.
All source data have been provided directly as supplementary tables or when too large, deposited on the manuscript Zenodo repo (DOI provided)

Research involving human participants, their data, or biological material

Policy information about studies with human participants or human data. See also policy information about sex, gender (identity/presentation), and sexual orientation and race, ethnicity and racism.

Reporting on sex and gender	Sex and gender analysis were not included in this study as it was irrelevant for the study hypothesis and query. In addition, we have a limited number of samples and all samples were used for a discovery analysis.
Reporting on race, ethnicity, or other socially relevant groupings	Race and ethnicity data were not available for the de-identified patient-derived samples used in this study and were therefore not included in the analysis.
Population characteristics	Glioblastoma tissues were surgically collected from patients recruited from St. Michael's Hospital (Toronto, Canada), Toronto Western Hospital (Toronto, Canada), The Hospital for Sickkids (Toronto, Canada), and the University of Calgary(Calgary, Canada)
Recruitment	Patients were collected with informed consent.
Ethics oversight	The study was approved under the Research Ethics Board at The Hospital for Sick Children (REB1000025582, REB0020010404), the University Health Network, the University of Calgary Ethics Review Board and the Health Research Ethics Board of Alberta, Cancer Committee and Arnie Charbonneau Cancer Institute Research Ethics Board (REB HREBA-CC-160762)

Note that full information on the approval of the study protocol must also be provided in the manuscript.

Field-specific reporting

Please select the one below that is the best fit for your research. If you are not sure, read the appropriate sections before making your selection.

☒ Life sciences ☐ Behavioural & social sciences ☐ Ecological, evolutionary & environmental sciences

For a reference copy of the document with all sections, see nature.com/documents/nr-reporting-summary-flat.pdf

Life sciences study design

All studies must disclose on these points even when the disclosure is negative.

Sample size	We used a total of 19 samples and these were selected based on if there were matched sets of phase contrast image datasets with bulk gene expression datasets. We used all available samples at the time of the study and hence the application of a statistical selection method was not applicable as datasets were scarce and limited.
Data exclusions	No data samples were excluded
Replication	We attempted to reproduce the original study where we used 15 samples with 4 additional new samples that were independent samples to the 15. We used used two different approaches - an unsupervised approach and a second linear regression model to test our hypothesis and both approaches resulted in overlapping results confirming our findings.
Randomization	This is not relevant to our study as we had limited number of samples.
Blinding	This is a discovery study and cohort and hence blinding is not applicable.

Reporting for specific materials, systems and methods

We require information from authors about some types of materials, experimental systems and methods used in many studies. Here, indicate whether each material, system or method listed is relevant to your study. If you are not sure if a list item applies to your research, read the appropriate section before selecting a response.

Materials & experimental systems

n/a	Involved in the study
☒	☐ Antibodies
☐	☒ Eukaryotic cell lines
☒	☐ Palaeontology and archaeology
☒	☐ Animals and other organisms
☒	☐ Clinical data
☒	☐ Dual use research of concern
☒	☐ Plants

Methods

n/a	Involved in the study
☒	☐ ChIP-seq
☒	☐ Flow cytometry
☒	☐ MRI-based neuroimaging

Eukaryotic cell lines

Policy information about cell lines and Sex and Gender in Research

Cell line source(s)	All cell lines were obtained from glioblastoma grade 4 patients.
Authentication	Authentication was done with PCR using a panel of polymorphic markers. The lines were matching the patients they were derived from.
Mycoplasma contamination	All lines were tested for mycoplasma contamination and were tested to be free of contamination.
Commonly misidentified lines (See ICLAC register)	NA

Plants

Seed stocks	NA
Novel plant genotypes	NA
Authentication	NA
